# Semantic Processing Impairment in Patients with Temporal Lobe Epilepsy

**DOI:** 10.1155/2015/746745

**Published:** 2015-07-16

**Authors:** Amanda G. Jaimes-Bautista, Mario Rodríguez-Camacho, Iris E. Martínez-Juárez, Yaneth Rodríguez-Agudelo

**Affiliations:** ^1^Proyecto de Neurociencias, Facultad de Estudios Superiores Iztacala, Universidad Nacional Autónoma de México, 54090 Ciudad de México, MEX, Mexico; ^2^Departamento de Neuropsicología, Instituto Nacional de Neurología y Neurocirugía, 14269 Ciudad de México, DF, Mexico; ^3^Clínica de Epilepsia, Instituto Nacional de Neurología y Neurocirugía, 14269 Ciudad de México, DF, Mexico

## Abstract

The impairment in episodic memory system is the best-known cognitive deficit in patients with temporal lobe epilepsy (TLE). Recent studies have shown evidence of semantic disorders, but they have been less studied than episodic memory. The semantic dysfunction in TLE has various cognitive manifestations, such as the presence of language disorders characterized by defects in naming, verbal fluency, or remote semantic information retrieval, which affects the ability of patients to interact with their surroundings. This paper is a review of recent research about the consequences of TLE on semantic processing, considering neuropsychological, electrophysiological, and neuroimaging findings, as well as the functional role of the hippocampus in semantic processing. The evidence from these studies shows disturbance of semantic memory in patients with TLE and supports the theory of declarative memory of the hippocampus. Functional neuroimaging studies show an inefficient compensatory functional reorganization of semantic networks and electrophysiological studies show a lack of N400 effect that could indicate that the deficit in semantic processing in patients with TLE could be due to a failure in the mechanisms of automatic access to lexicon.

## 1. Introduction

Temporal lobe epilepsy (TLE) constitutes 80% of focal epilepsies and is the most frequent form in adults [1, 2]. Mesial TLE with hippocampal sclerosis is an epileptic syndrome very resistant to pharmacologic treatment [3], and approximately 50% of patients with this form of epilepsy present drug resistance, so it has been considered both a medical and a social problem [4].

Patients with TLE show great heterogeneity in clinical and cognitive characteristics. It is known that these patients are at significant risk for cognitive impairment and behavioral abnormalities [5–7]. Impairment of the memory system constitutes the most common cognitive deficit in TLE; around 70% of patients have problems in episodic memory, associated with the presence of hippocampal sclerosis [8, 9].

Neuropsychological studies have reported alterations in episodic memory with profiles of lateralization of selecting deficits in the verbal memory of patients with left TLE and in the visual memory of patients with right TLE [10–18]. It is interesting that recent research has also found impairment in the semantic memory of patients with TLE [19–24]; however, there are still few reports that have explored these alterations in detail.

Semantic memory is important because it contains the knowledge that allows individuals to communicate, represent, and mentally operate situations, objects, and relations with the world, which otherwise are not available to the senses. It allows the identification of events and use of general knowledge that forms the basis of our knowledge of the world [25]. The impairment of this kind of memory is manifested by difficulties in naming and concept definition and by poor understanding of oral or written language, and it can also impact other cognitive functions [26].

The main objective of this paper is to review recent research regarding the consequences of TLE on semantic memory, considering neuropsychological, electrophysiological, and neuroimaging findings. Taking into consideration the important relation between the hippocampus and TLE, an additional objective is to analyze the role of this cerebral structure in the semantic processing.

The literature included in this review was published between 2000 and 2014 for neuroimaging and electrophysiological studies and between 1990 and 2014 for neuropsychological studies. The search was made in PubMed using as key words: temporal lobe epilepsy, hippocampus, semantic memory and semantic processing, event related potentials, and N400 component and functional magnetic resonance imaging.

## 2. Semantic Memory

Memory is an active cognitive process that involves the acquisition, storage, and retrieval of information. Acquisition is achieved through coding, which is the initial process by which physical information is transformed into a mental representation. Coding takes various forms that depend on the characteristics of the stimulus; it is carried out from the level of processing of physical and sensory traits to the most abstract and semantic of information [27]. Storage is the ability to accumulate and maintain previously registered information for a period of time. Retrieval implies access, search, and extraction of information held in different kinds of storage [28].

Semantic memory is integrated by knowledge acquired about the world, including word meanings, kinds of information, events, and ideas. It represents organized mental knowledge about words and other verbal symbols, their meanings, and referents, about relations around them, and about the rules, formulas, and/or algorithm for the manipulation of these symbols, concepts, and relations. Therefore, it is the memory necessary for language [29].

### 2.1. Searching for the Neuroanatomy of Semantic Memory

Studies of patients with amnesia reported the following neuroanatomical structures related to memory: anterior and medial part of the temporal lobe, prefrontal region, portions of the limbic system including the hippocampal gyrus and uncus, thalamic nuclei, and mammillary bodies [30].

Squire et al. in 2004 [31] suggested that the medial temporal lobe includes a system of anatomically related subcortical structures that are critical for declarative memory; these structures along with the neocortex operate to establish and maintain long-term memory. Subcortical structures system is constituted by the hippocampal region (CA1–3 fields, dentate gyrus, and subicular complex), the adjacent perirhinal cortex, the entorhinal cortex, and parahippocampal cortex. Actually Squire et al. (1993) [32] proposed that the semantic memory depends on the integrity of the hippocampus and the neuroanatomical structures related to it, such as the medial temporal lobe and diencephalon.

The anterior temporal lobe has also been related to semantic memory; however, the role of this structure is unclear because patients with focal damage have deficiencies in recognizing and naming people that are famous or familiar, suggesting that this area might store specific semantic information; nonetheless, epileptic patients with lobectomy in this area do not show significant alterations in semantic memory [33].

Recent functional magnetic resonance image (fMRI) studies have added some more specific information on the matter. The anterior inferior and medial temporal gyrus, the anterior fusiform gyrus, and the upper anterior temporal sulcus were activated in normal subjects performing semantic tasks. It was proposed that such structures are importantly linked to semantic memory [34, 35]. Medial temporal gyrus integrates auditory and visual information and the anterior ventral surface produces transmodals (i.e., more integrated and abstract forms of objects), representing a core structure for the formation of coherent concepts.

Binder and Desai [36] proposed a neuroanatomical model of semantic memory based on data from fMRI of healthy participants and subjects with impaired memory. This model related the cortices of specific modality with the temporoparietal supramodal cortices which store more abstract representations of knowledge of data and the prefrontal cortex that controls the activation and selects behavior directed towards a goal. On the other hand, the posterior cingulate gyrus and the precuneus may function as an interface between the semantic network and the hippocampal memory system, which could help to codify significant events in episodic memory.

In summary, clinical data show different structures involved in semantic memory, among which are the temporoparietal cortex, left anterolateral temporal cortex, the medial and inferior temporal gyrus, fusiform gyrus, the amygdala, ventromedial frontal cortex, perirhinal cortex, and inferior frontal cortex. These structures have been related to semantic memory because different brain lesions that result in impaired memory provide evidence as which brain areas are related to different types of memory, but since the lesions are not circumscribed, it is difficult to determine whether a specific deficit is the result of a lesion to a specific structure [37].

### 2.2. The Hippocampus and Its Relation with Semantic Memory

The functional role of the hippocampus in semantic processing is currently under debate and there are two points of view about it.

On one side, the* episodic theory* of the hippocampus suggests that it plays a selective role in episodic memory but contributes very little or nothing to semantic memory [38]. The main evidence that supports this position comes from the cognitive description of a group of patients with development amnesia, who during their first years of life acquire damage to the hippocampus (secondary to perinatal hypoxic-ischaemic) and as a consequence, they exhibit impaired episodic memory with intact or spare semantic memory [39, 40].

On the other side, the* declarative theory* suggests that the hippocampus, together with the entorhinal, perirhinal, and parahippocampal cortices, contributes to both episodic memory and semantic memory [41]. This position is based on evidence from cognitive results in patients with amnesia due to acquired pathologies at adulthood in which the lesion of the mesial temporal cortex produced impairment in both types of memory [42–45]. In addition research with functional neuroimaging in healthy subjects has described hippocampal activation during retrieval of various kinds of semantic knowledge, such as retrieval of elements of semantic categories [46], semantic decisions [47, 48], historic events [49], and famous people [50, 51].

Until now, research has suggested that in the adult brain the hippocampus plays an important role in both episodic memory and semantic memory, since both are significantly impaired following even discrete damage limited to the hippocampus. When the damage is sustained at an early age, such as development amnesia, it is possible that there is a certain functional reorganization in the damaged temporal lobe [41].

## 3. Semantic Memory and Temporal Lobe Epilepsy

From the case of patient H.M. published by Scoville and Milner [52], the study of TLE has contributed importantly to knowledge about the neurocognition of human memory.

### 3.1. Neuropsychological Studies

Memory is the most studied cognitive process and one where more abnormalities have consistently been found in patients with TLE.

Regarding semantic memory, the majority of the studies in patients with TLE have analyzed their performance in verbal fluency and naming tasks, finding that those patients with seizures in the dominant temporal lobe for language (generally left) show deficits characterized by naming failures and poor verbal fluency [19, 22, 24, 53–55].

The effect of mesial TLE on semantic verbal fluency has been found to depend greatly on the interaction between hippocampal sclerosis (HS) and the laterality of the epileptic focus. Gleissner and Elger [21] compared the performance of TLE patients based on the type of lesion (HS versus extrahippocampal lesions such as tumors or arteriovenous malformations) and the laterality of the focus. They found that verbal fluency was impaired regardless of the laterality of the focus in patients with TLE and HS, unlike patients with other types of lesions. Patients with left TLE had deficits independent of the type of lesion, while only patients with right TLE along with HS exhibited deficits. Based on these results, the authors concluded that the hippocampus plays an important role in the retrieval of semantic knowledge; however, independently of the presence of HS, damage to the dominant hemisphere for language is sufficient to affect verbal fluency.

Only two neuropsychological studies have evaluated the semantic system extensively, through the use of a greater number of tests, including both verbal and nonverbal stimuli, and exploring the relation between abnormalities and the laterality of the epileptic focus. Both Giovagnoli et al. [20] and Messas et al. [23] demonstrated deficits in semantic memory in patients with TLE; however, the former reported that only patients with left TLE had deficits in retrieving both verbal and nonverbal semantic information [20] while the latter found that patients with either left or right TLE had deficits in the semantic system, although the most pronounced and extensive problems were in patients with left TLE [23].

Studies have also been carried out to explore the semantic aspect of remote memory of patients with TLE [56–59]. In these studies questions about events of public knowledge corresponding to a specific period and related to politics, sports, scandals, social events, or catastrophes were made; photos of famous people faces that had to be recognized and identified (e.g., to give the name, occupation, or say if they were alive or dead) were displayed.

The results of these studies were that patients with TLE had problems retrieving names, a reduction in conceptual knowledge regarding famous people, and failures both in spontaneous evocation and recognition of events. Specifically, patients with left TLE had a poor performance compared to patients with right TLE in the information retrieval about public events and exhibit a selective impairment characterized by a temporal gradient, with poor naming of faces from the most recent periods compared to distant ones. Patients with right TLE showed impairment in the recognition, identification, and naming of famous faces [56–59].

Neuropsychological studies have offered sufficient evidence of the presence of impairment in semantic memory in patients with TLE and it seems that lateralized lesions in the temporal lobe have differential effects on the retrieval of semantic information. However, it is important to consider that the majority of the tests used to assess this type of memory also require the participation of other cognitive processes such as attention and executive functions, which are also altered in these patients [60–64]. Therefore, there is a possibility that semantic dysfunction in TLE may be secondary to deficits in other cognitive processes [20].

### 3.2. Event-Related Potentials (ERP) Studies

ERPs allow an online observation of the cognitive processes that generate the observed behavior by reflecting changes in cerebral electrical activity that keep a specific temporal relationship with the physical stimuli and the cognitive processes that provoke them.

ERPs greatest advantage is a real-time assessment (in milliseconds) of electrical activity and cognitive processes in the brain, offering high-resolution information concerning chronology and sequence of cognitive processes. They also allow distinguishing in which stage of information processing an alteration can be found [65].

Study through ERP of patients with TLE candidates for surgical treatment offers a unique opportunity to know the electrophysiological correlates of cognitive processes. In these patients the electrodes can be placed directly on relevant structures for language or memory and so correlate deficits of ERP in paradigms associated with these processes.

Therefore, more studies through intracranial records have been carried out than noninvasive studies; however, the majority has focused on episodic memory [66–70].

In spite of the advantages this kind of study offers, there are also some problems. On one hand, the results are limited to plausible epileptogenic areas, so anatomic coverage restriction limits the information about other areas. On the other hand, invasive records are only used in patients undergoing a temporal lobectomy procedure, so the data obtained cannot be contrasted with healthy people that serve as a parameter of normality, restricting the generalization of findings.

The scarce noninvasive studies corroborate the effect of TLE on semantic processing. Olichney et al. [71] carried out a research to investigate if impairment in the N400, ERP component related to semantic processing, was related to the laterality of the epileptic focus. They used a crossmodal semantic categorization task; with the auditory presentation of sentences followed by target words presented visually, 50% were congruent and 50% were incongruent regarding the previously presented category (e.g., a type of wood, cedar/pancake). Patients with right TLE showed an increase in the amplitude of N400 for incongruent words compared with congruent (N400 effect). In contrast, patients with left TLE did not show this N400 effect.

The results of this study suggest that semantic processing is sensitive to left temporal dysfunction; however, the question remains open as to whether the absence of the N400 effect may be associated with deficits in access and retrieval of semantic information or with the attentional processes necessary to carry out the semantic categorization task.

Miyamoto et al. [72] investigated the mechanism of semantic priming (facilitation effect of the processing of semantically related words) in patients with TLE and a control group of healthy participants through a visual task of category matching. Each trial of the task was composed of a warning sign, a prime (first word), and a target (second word). Two conditions were presented according to the semantic relationship between the prime and the target: (1) match condition (both stimuli belonged to the same category, such as sparrow-bird) or (2) mismatch condition, where the target and the prime belonged to different semantic categories (such as dragonfly-fish). Data were analyzed at the behavioral level (reaction time and percentage of errors) and at the electrophysiological level through the amplitude of N400, contingent negative variation (CNV), and late positive component (LPC), the two latter being related to different demands on attention processing.

The behavioral results showed that patients with TLE had a nonsignificant tendency to present more prolonged reaction times and a greater percentage of errors for both conditions compared to the control group; nevertheless, both groups had shorter reaction times in the related condition compared with the nonrelated, revealing a behavioral semantic priming effect. Regarding electrophysiological data, a reduction was found in the amplitude of N400 component for both conditions (match and mismatch) in patients compared with the control group, in addition to an absence of the N400 effect (lack of increase in amplitude in mismatch compared with match condition). On the other hand, there was no intergroup difference in the amplitude of CNV and LPC components. The authors concluded that the reduced amplitude of N400 for both conditions in the patients suggested the presence of an alteration in generators of this component (located in the left temporal pole). They stated that failures in semantic processing can be located at the level of access and automatic activation of semantic information and that deficits cannot be attributed to attention disorders given that the CNV and LPC components did not show abnormality.

Jaimes-Bautista et al. [73] presented preliminary results that corroborated these findings. They carried out a study to determine if semantic impairment in TLE was related to deficit in automatic activation of semantic networks or to failures in strategies for information retrieval. A lexical decision tasks were used, manipulating the interstimuli interval (ISI) to generate automatic and controlled semantic priming. The results showed that TLE patients did not present the N400 effect associated with semantic priming, for both the automatic and controlled conditions. This study showed that semantic processing impairment in patients with TLE is related to deficiencies in the automatic activation mechanisms, and in addition, it seems that patients do not benefit from the use of strategies for retrieval of information.

Although the studies with noninvasive ERP in patients with TLE are scarce, they show potential to reveal the underlying mechanisms involved in the semantic memory processes impairment in these patients.

### 3.3. Functional Magnetic Resonance Images (fMRI) Studies

In TLE, electrical hyperexcitability is spread within the temporal medial/limbic network, which includes the hippocampus, amygdala, entorhinal cortex, lateral temporal cortex, and extratemporal components such as the medial thalamus and inferior frontal lobes. These anatomical structures operate together to culminate in the eventual expression of seizures [74]. Various regions that form part of this network are also directly involved in semantic processing.

The results of researches into semantic processing in TLE through fMRI, using tasks of semantic decision (SDT), lexical decision (LDT), and verbal fluency (VFT), have demonstrated that patterns of cerebral activation in patients with TLE differ significantly from those of healthy subjects.

Köylü et al. [75] investigated the impact of mesial TLE on the network of frontal and temporal structures involved in semantic memory. They used auditory SDT that consisted of deciding if the objects presented could be found in a supermarket and judging their cost. Patients with TLE with either right or left epileptic focus had a lower percentage of correct answers compared to a control group of healthy participants. The results showed that the networks involved in semantic processing of patients differed significantly from the control group and, in addition, they showed a pattern of activation dependent on the side of the epileptic focus. Therefore, for the control group the pattern of activation included the bilateral frontal and temporal areas, with left predominance; in patients with left TLE activation was predominant in right frontal, bilateral temporal, and basal ganglia regions, and in patients with right TLE the pattern of activation showed a posterior network including temporal and parietal regions (lateral and mesial), with left predominance, as well as occipital regions, but without including frontal or subcortical areas.

These data show that both epileptic activity originating in the temporal lobe and the side of epileptic focus were associated with an alteration in the underlying neuronal circuits in semantic processing; in addition, not only the cortical but also subcortical structures appear to participate in semantic processing in TLE, particularly in the left hemisphere.

Bartha et al. [76] analyzed the contribution of hippocampal formation to semantic processing through the same SDT used by Köylü et al. [75]. During the task, both the control group and patients with TLE showed activation of bilateral hippocampal formation with left predominance. However, there were significant differences in the pattern of activation between groups. Patients with left TLE showed reduction in activation of the bilateral hippocampal formation compared with the control group and less activation of the right side compared with the right TLE group. Patients with right TLE activated the right hippocampal formation to a lesser degree but compared with the control group they showed an increase in left activation. The authors noted that the decrease in activation of the ipsilateral hippocampal formation in patients with left and right TLE was related to the reduction in hippocampus volume.

These results suggest that semantic processing is related to the bilateral functioning of the hippocampus and, while patients with right TLE appear to achieve compensation of the impairment through greater work by the contralateral hippocampus, patients with left TLE do not achieve the same kind of functional reorganization.

The above findings have been replicated in similar studies with different semantic tasks. Bonelli et al. [77] investigated the relation between the naming process and the integrity of semantic networks in patients with TLE through a VFT. In the control group and in patients with right TLE, activation of the left hippocampus during the VFT correlated positively with performance on naming task. In left TLE patients, the correlation was found with the activation of the left medial and inferior frontal cortex (and to a lesser degree, the right), which suggests compensatory strategies to support the naming process. In addition, they observed that a poor naming ability was parallel to the lack of left hippocampal activation.

It is then possible that the previously mentioned cerebral areas are recruited when the hippocampus does not function correctly, as in hippocampal sclerosis. In this way, the difficulties in the naming processes in patients with left TLE could be explained by the participation of a compensatory network in the frontal lobe, which turns out to be less functional.

Using an LDT (in which participants must decide if the stimulus presented is a word or not), Jensen et al. [78] also analyzed if the presence of hippocampal sclerosis in patients with left TLE related to the efficiency of semantic processing and the associated cerebral activation. Patients with hippocampal sclerosis showed significantly longer reaction times than the control group. Neuroimaging data revealed greater activation in the inferior and medial frontal gyrus and the precuneus bilaterally during the LDT in the control group; the group of patients without hippocampal sclerosis showed greater activation only in the left medial temporal gyrus, while the group with hippocampal sclerosis showed greater activation in various regions, such as the superior and medial temporal cortex, precuneus and left cingulated gyrus, and right medial temporal and supramarginal cortex.

These results again corroborate that when the hippocampal sclerosis is in the left temporal lobe, various cerebral structures are recruited but they do not achieve sufficient compensation, so deficient semantic processing is presented.

Studies with tractography have shown that TLE is associated with impairment in the integrity of the frontotemporal connections, describing a reduction in the structural connections in the epileptogenic hemisphere and a possible increase in compensatory connections in the unaffected contralateral hemisphere [79, 80]. Therefore, deficiency in frontotemporal connectivity may affect function in this part of the semantic memory network.

Neuroimaging studies suggest the possibility that the morphofunctional bases that give rise to semantic impairment in TLE may be hippocampal sclerosis or the epileptic activity in course and its propagation to other temporal and frontal regions.

## 4. Conclusions

The objective of this paper was to present an updated review of research regarding the consequences of TLE on semantic memory and to analyze the role of the hippocampus in semantic processing.

Each of the methods reviewed contribute to approaching the subject from different levels of analysis, allowing a complementary vision. Neuropsychology offers a general view concerning semantic deficits at the behavioral level, while the ERP reveal specific data about the stage in which information processing is impaired and neuroimaging studies allow knowing the cerebral structures involved in semantic dysfunction.

Neuropsychological and functional neuroimaging studies have investigated the effect of alteration of the hippocampus and the laterality of epileptic focus on semantic processing. The results show that the hippocampus has an important participation in semantic processing, supporting the theory of declarative memory of the hippocampus. It has also been demonstrated that when the lesion in the temporal lobe is in the dominant hemisphere for language functioning, semantic processing is altered independently of the type of lesion (i.e., hippocampal sclerosis, tumors, or arteriovenous malformations).

Additionally, studies with fMRI show that TLE is associated with deficits in the functional organization of cortical networks involved in semantic processing, likely caused by morphological changes inherent to chronic TLE, such as hippocampal sclerosis. Therefore, during semantic tasks, TLE relates to a pattern of activation that is different from the normal, probably due to a compensatory functional reorganization that includes various cerebral structures, that nevertheless is less functional.

In spite of the aforementioned findings, the underlying mechanisms of semantic processing impairment in TLE are still not completely understood; that is, it is still not clear if it is due to failures in accessing and retrieving information from semantic storage or even degradation of such information. Some studies have suggested the possibility that semantic deficit may be secondary to alterations in other cognitive processes, since the majority of the experimental tasks and neuropsychological tests used to evaluate semantic memory require the participation of other processes such as attention and executive functions, which are also affected by this disease.

Studies through ERPs have offered data that allow resolution of this question. The specific finding of reduction in the amplitude of the N400 component and the lack of N400 effect, added to the fact that ERP components that reflect other cognitive processes are not affected, seems to indicate that the deficits are directly related to access and retrieval of information from semantic storage. Nevertheless, ERP studies on the effect of TLE on semantic memory are scarce and it is necessary to develop more research in this area.

Future studies with the methods currently available to neuroscience may approach aspects such as the following: if semantic impairment is exclusively linguistic or is also presented at nonlinguistic level and if it is related to the laterality of epileptic focus.

Another important issue to analyze is the effect of various clinical variables such as age at onset, chronicity, frequency of seizures, and antiepileptic drugs on semantic processing.

These aspects, among other questions, would be of great utility in the development of knowledge of semantic impairment in TLE, as well as of the cerebral structures and mechanisms involved in the semantic memory functioning.

On the other hand, greater knowledge of the memory impairments in TLE patients will allow implementing more specific programs for cognitive rehabilitation for these patients.

TLE, compared with other kinds of pathologies such as dementia, traumatic brain injury, and cerebral vascular disease, allows the study of a younger clinical population with known and more easily identifiable lesion. For this reason, patients with TLE, especially those with hippocampal sclerosis, offer a unique clinical scenario to study the consequences that mesial temporal lobe damage may have on memory systems. In this way, TLE continues to serve as an important model for understanding the cerebral bases of memory.
